# Doxapram as an additive to propofol sedation for endoscopic retrograde cholangiopancreatography: a placebo-controlled, randomized, double-blinded study

**DOI:** 10.1007/s00464-019-07344-2

**Published:** 2020-01-28

**Authors:** Jarno Jokelainen, Anna Belozerskikh, Harri Mustonen, Marianne Udd, Leena Kylänpää, Outi Lindström, Maxim Mazanikov, R. Pöyhiä

**Affiliations:** 1 Department of Anesthesia and Intensive Care Medicine, South Karelia Central Hospital, Valto Käkelän katu 1, 53130 Lappeenranta, Finland; 2 University of Helsinki, Helsinki, Finland; 3 Department of Anesthesia and Intensive Care Medicine, Helsinki University Central Hospital, Helsinki, Finland; 4 Department of Gastroenterological and General Surgery, Helsinki University Hospital, Helsinki, Finland; 5 Kauniala Hospital, Kauniainen, Finland

**Keywords:** Endoscopy, Sedation, Cholangiopancreatography, Endoscopic, Doxapram, Propofol

## Abstract

**Background:**

Endoscopic retrograde cholangiopancreatography (ERCP) requires moderate to deep sedation, usually with propofol. Adverse effects of propofol sedation are relatively common, such as respiratory and cardiovascular depression. This study was conducted to determine if doxapram, a respiratory stimulant, could be used to reduce the incidence of respiratory depression.

**Methods:**

This is a single-center, prospective randomized double-blind study performed in the endoscopy unit of Helsinki University Central Hospital. 56 patients were randomized in a 1:1 ratio to either receive doxapram as an initial 1 mg/kg bolus and an infusion of 1 mg/kg/h (group DOX) or placebo (group P) during propofol sedation for ERCP. Main outcome measures were apneic episodes and hypoxemia (SpO_2_ < 90%). Mann–Whitney test for continuous variables and Fisher’s exact test for discrete variables were used and mixed effects modeling to take into account repeated measurements on the same subject and comparing both changes within a group as a function of time and between the groups.

**Results:**

There were no statistically significant differences in apneic episodes (*p* = 0.18) or hypoxemia (*p* = 0.53) between the groups. There was a statistically significant rise in etCO_2_ levels in both groups, but the rise was smaller in group P. There was a statistically significant rise in Bispectral Index (*p* = 0.002) but not modified Observer’s Assessment of Agitation/Sedation (*p* = 0.21) in group P. There were no statistically significant differences in any other measured parameters.

**Conclusions:**

Doxapram was not effective in reducing respiratory depression caused by deep propofol sedation during ERCP. Further studies are warranted using different sedation protocols and dosing regimens.

**Clinical trial registration** ClinicalTrials.gov ID NCT02171910.

Endoscopic retrograde cholangiopancreatography (ERCP) is a very demanding endoscopic procedure that usually cannot be performed without deep sedation or general anesthesia because of substantial procedural discomfort and pain [1–3]. There is no agreement on the best method of anesthetic care for ERCP. Traditionally, benzodiazepines and opioids were used but propofol has been gaining popularity around the world for the past few decades [4]. Propofol sedation is not without risks [5], one of the most important of which is respiratory depression and hypoxemia.

Doxapram (1-ethyl-4-(2-morpholin-4-ylethyl)-3,3-diphenyl-pyrrolidin-2-one), a central and peripheral respiratory stimulant and a non-specific stimulant of the central nervous system, has been widely used to reverse respiratory depression [6, 7]. Doxapram has recently been shown to shorten the time to spontaneous breathing after total intravenous anesthesia using propofol and remifentanil [8]. To our knowledge, there are only a few studies [9–12] done on humans on the effect of doxapram during sedation, but none of them investigated the use of doxapram with propofol during endoscopic sedation.

In our preliminary tests, doxapram showed promising results. However, these tests were not randomized or blinded. Therefore, this study was carried out to assess the efficacy of doxapram as an additive to deep propofol sedation in reducing the incidence of respiratory depression in a randomized double-blinded protocol. The study was conducted in the endoscopic unit of Meilahti Hospital, a tertiary university clinic, where over 1200 ERCP procedures are performed annually.

## Materials and methods

This study was approved by the institutional Ethics Committee of Helsinki University Central Hospital (Ethics Committee, Department of Surgery, Biomedicum Helsinki 2 C, Tukholmankatu 8 C, PL 705, 00029 HUS, Finland. DNRO 281/13/03/2013) on January 22, 2014. This study was registered in The EudraCT system (EudraCT 2013-003873-85) and approved by the Finnish Medicines Agency on April 12, 2016. The study was also registered in the ClinicalTrials.gov registry (ClinicalTrials.gov ID NCT02171910).

According to a power analysis performed before the study, at least 18 patients per group were required to detect a 30% difference in respiratory depression between the groups (*β* = 0.1, *α* < 0.05). A total of 56 patients scheduled for an ERCP procedure from November to December 2016 were enrolled in the study. Exclusion criteria were age > 75, epilepsy, coronary artery disease (stable or unstable angina pectoris), chronic obstructive pulmonary disease, acute alcohol withdrawal syndrome, allergy to propofol, or doxapram.

Patients were randomized at the ratio of 1:1 into two groups, according to a computer-generated table of random numbers to receive doxapram (group DOX) or placebo (group P) in a double-blind manner. The patient and the anesthesiologist who was also responsible for the data collection were blinded to the study drug administered.

Propofol (10 mg/ml) infusion was given 0.5 ml/kg/h (83.3 µg/kg/min). Before the infusion, the patients’ pharynx received topical anesthesia with lidocaine spray (Xylocaine 10 mg/dos®, 5 sprays), alfentanil 0.5 mg i.v., glycopyrronium 0.2 mg i.v., and lidocaine 20 mg i.v. Endoscope was inserted once Bispectral index (BiS) was < 60. In the group DOX, the patient received an initial bolus of doxapram 10 mg/ml 0.1 ml/kg and an infusion of doxapram 10 mg/ml at 0.1 ml/kg/h. The group P received an initial bolus on 9 g/l NaCl solution 0.1 ml/kg and an infusion of 9 g/l NaCl solution at 0.1 ml/kg/h. During the procedure, propofol infusion was adjusted by 10 ml/h increments in order to keep BiS < 60 or modified Observer’s Assessment of Alertness/Sedation (mOAAS) at level 1–2.

If peripheral oxygen saturation (SpO_2_) was < 88% or if the patient stopped breathing, patients in both groups were given doxapram 1 mg/kg i.v. openly. The dose could be repeated if needed. If breathing would not start despite doxapram, the procedure was to be paused and mask ventilation commenced. After the patient started breathing again, the procedure could continue, and sedation would be continued using propofol boluses of 10–20 mg i.v. and the study would be stopped for the patient in question.

The following data of each patient were registered in a prospective manner: age, weight, height, American Society of Anesthesiology physical status classification (ASA), performed procedures, duration of the procedure, consumption of propofol, doxapram, or placebo. Heart rate, rate of breathing, SpO_2_, end-tidal CO_2_ (etCO_2_), and non-invasive blood pressure (NIBP) were recorded at 5-min intervals during the procedure. The level of sedation was assessed using BiS and mOAAS at 5-min intervals during the procedure [13]. Use of phenylephrine or atropine was recorded. The satisfaction of the endoscopist to the sedation (ease of inserting the endoscope, patient co-operation (low number when lightly sedated (optimally), high when deeply sedated by definition), gagging, coughing, belching, distracting movement by the patient using a 4 step scale from none to plenty, and difficulty of the procedure by The Schutz scale [14] was recorded.

During recovery, patient vital signs (heart rate, SpO_2_, non-invasive blood pressure), pain intensity (verbal rating scale, 0 = no pain and 4 = severe pain), sedation level (Gillham score) [15], and recovery rapidity (Aldrete score) [16, 17] were registered at 5-min intervals until discharge. Postprocedural nausea, if present, was treated and registered. Before discharge from the recovery room, patients were asked about their satisfaction with the sedation. A seven-step numeric scale (1 = very unsatisfied, 7 = very satisfied) was applied for the measurement of patient satisfaction with sedation [18]. An Aldrete score of ≥ 9 and mild pain as the maximum were defined as discharge criteria.

Main outcome measures were apneic episodes and hypoxemia defined as SpO_2_ < 90%. Secondary outcome measures were as follows:

1. SpO_2_, blood pressure, heart rate, rate of breathing and end-tidal CO_2_, BiS and mOAAS, during the procedure,
2. blood pressure, heart rate, rate of breathing, pain intensity, Gilham score, and Aldrete score during recovery,
3. patient and endoscopist satisfaction.

The results are reported as mean and standard deviation (mean [SD]) or median and interquartile range (IQR). The possible differences between groups were tested with the Mann–Whitney test for continuous variables and with the Fisher’s exact test for discrete variables. Mixed effects modeling was used to take into account repeated measurements on the same subject and comparing results between the groups. Linear model was used for continuous variables and multinomial logistic regression model for ordinal variables. Both changes within a group as a function of time and differences between the groups were taken into account. There were enough data points for statistical analysis from the beginning of the procedure to 30 min into the procedure. Groups were also analyzed in separate mixed effects model to obtain the possible linear time dependency within the group. Statistical calculations were generated using IBM SPSS Statistics 24 (International Business Machines Corporation, Endicott, NY, USA).

## Results

**Fig. 1:**
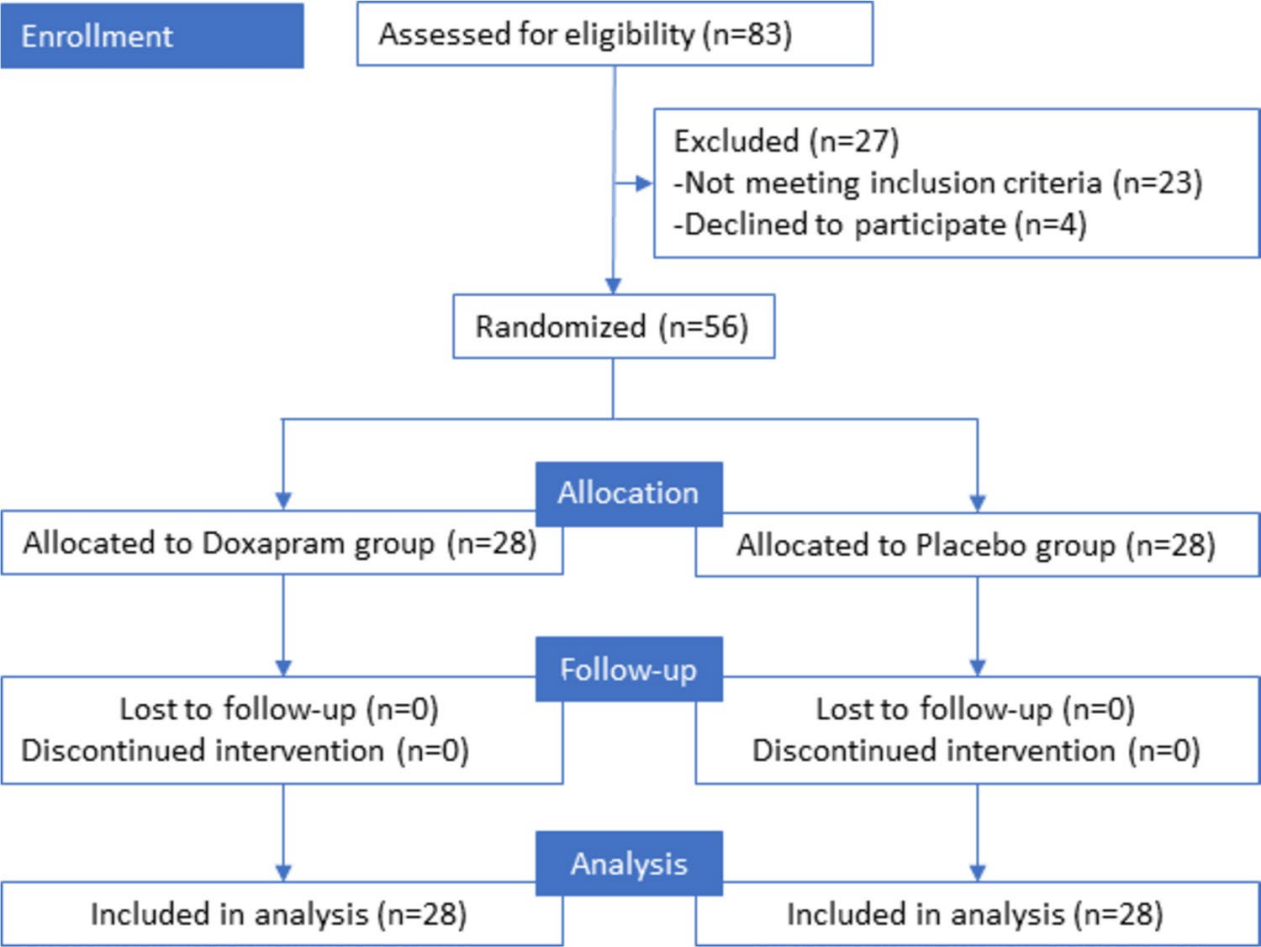
Consort 2010 flow diagram

A total of 56 patients were recruited to the study. A CONSORT diagram is presented in Fig. 1. Demographics, drug consumption, and ERCP details are shown in Table 1. There were no statistically significant differences between the groups.

**Table 1:** Demographics, propofol consumption, ERCP indications, and performed procedures in patients receiving doxapram or placebo

	Doxapram (*n* = 28)	Placebo (*n* = 28)	*p*
Age; years median (range)	51 (19–70)	48 (20–68)	0.93
Male/female	17(51%)/11 (39%)	16 (57%)/12 (43%)	1.0
BMI kg/m^2^ (median, range)	23.9 (14.7–33)	25.7 (18–34.6)	0.21
Length of the procedure min (median, range)	22 (3–52)	20 (5–51)	0.81
ASA I–II	21 (75%)	20 (71%)	0.76
ASA III–IV	7 (25%)	8 (29%)	
Propofol consumption, milligrams	368.35 (229.56)	353.83 (116.16)	0.40
procedure duration, minutes median (IQR)	22.71 (13.70)	23.43 (10.91)	0.63
Patient satisfaction, median (IQR)	6.57 (0.50)	6.67 (0.55)	0.21
Endoscopist satisfaction, median (IQR)	7.29 (1.46)	7.14 (1.24)	0.26
ERCP indication^a^			
Common bile duct stones	0	3 (10%)	0.24
Biliary stricture	4 (14%)	6 (21%)	0.73
Primary sclerosing cholangitis	15 (54%)	10 (32%)	0.18
Postoperative biliary leak	1 (4%)	1 (4%)	1.0
Liver transplantation and stricture	1 (4%)	2 (7%)	1.0
Chronic pancreatitis/pseudocyst	9 (32%)	6 (21%)	0.33
Procedure^a^			
Biliary cytology	17 (61%)	13 (46%)	0.28
Biliary sphincterotomy	7 (25%)	6 (21%)	0.75
CBD stone extraction	0	3(10%)	0.24
Biliary dilatation	4 (14%)	4 (14%)	1.0
Biliary stent application, exchange or removal	6 (21%)	5 (17%)	1.0
Pancreatic sphincterotomy	2 (7%)	1 (4%)	0.53
Pancreatic cytology	1 (4%)	1 (4%)	1.0
Pancreatic dilatation	6 (21%)	1 (4%)	0.10
Pancreatic stent application, exchange or removal	9 (32%)	6 (21%)	0.54
Pseudocystogastrostomy/duodenostomy	0	1 (4%)	1.0
Single operator cholangioscopy	0	2 (7%)	0.49
ERCP degree of difficulty			0.266
I	9 (32%)	11 (39%)	
II	7 (25%)	5 (18%)	
III	12 (43%)	9 (32%)	
IV	0	3 (11%)	

^a^Patient may have several indications and procedures

*ASA* American Society of Anesthesiologists physical status classification system, *BMI* Body Mass Index, *CBD* common bile duct

### Primary outcome measures

Seventeen patients in group P and 11 patients in group DOX had apneic episodes. There were no statistically significant differences between the groups (*p* = 0.18). Hypoxemia was recorded on 5 patients in group P and 8 patients in group DOX, with no statistically significant difference (*p* = 0.53). The procedure had to be paused once in group DOX because of hypoxemia, and the procedure was delayed once for the same reason after induction of sedation. Both times mask ventilation was required. There was no need for mask ventilation or pauses in procedure in group P. The amount of doxapram given as infusion in the DOX group was 113.8 (34.1) mg, and additional doxapram boluses were given 3 times in group P and once in group DOX, with no statistically significant difference (*p* = 0.61).

### Secondary outcome measures

**Fig. 2:**
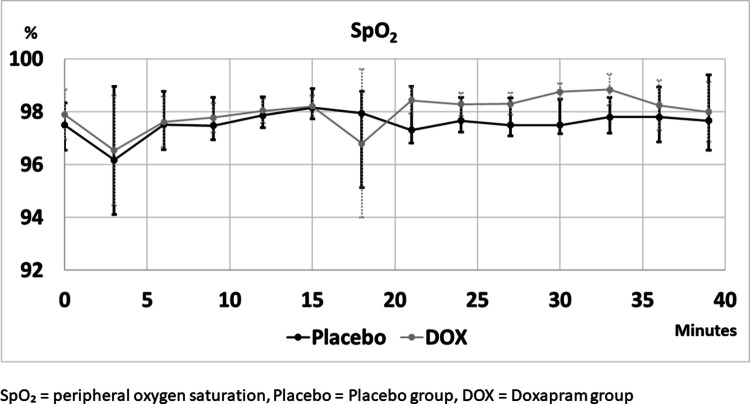
Peripheral oxygen saturation levels during ERCP

**Fig. 3:**
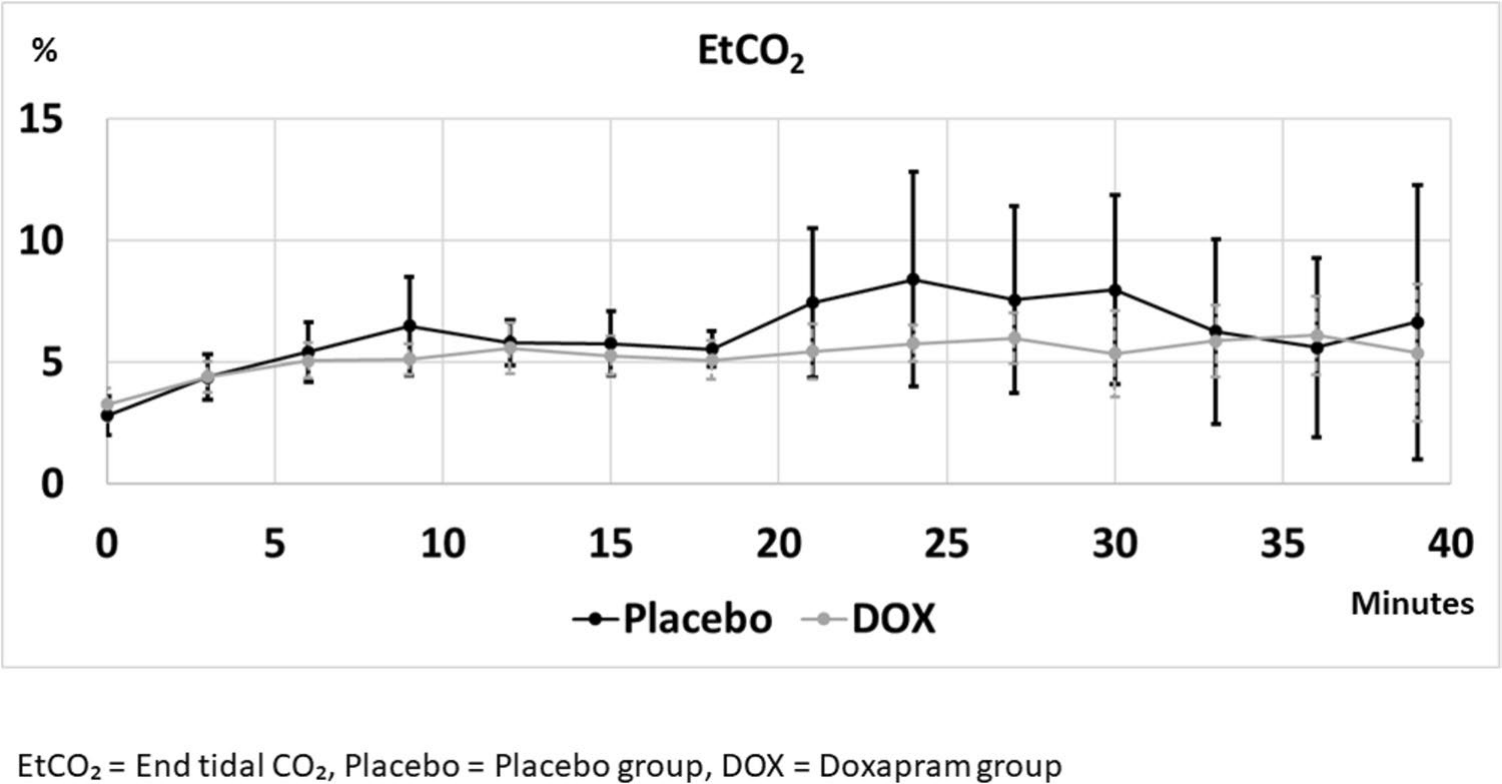
End-tidal CO2 levels during ERCP

SpO_2_ and etCO_2_ levels during procedure are shown in Figs. 2 and 3 respectively. Results of the mixed effects model-to-model linear time dependence analysis of secondary outcome measures during the procedure are shown in Table 2. There was a statistically significant rise in etCO_2_ levels in both groups, and the rise was smaller in group P than in doxapram group (Table 3). There was a statistically significant rise in BiS in both groups (Table 2) but not mOAAS.

**Table 2:** Mixed effects model-to-model linear time dependence of measured variables in different groups during ERCP

Group	Measurement	*B* (time)	95% CI		*p*
			Lower	Upper	
P	Heart rate	0.45	0.16	0.73	0.002
Dox	Heart rate	0.27	0.09	0.45	0.004
P	Breath rate	0.28	0.18	0.38	< 0.001
Dox	Breath rate	0.29	0.18	0.40	< 0.001
P	SpO_2_	0.02	− 0.03	0.07	0.515
Dox	SpO_2_	0.04	− 0.01	0.09	0.14
P	BIS	− 0.51	− 0.78	− 0.24	< 0.001
Dox	BIS	− 0.76	− 1.11	− 0.40	< 0.001
P	EtCO_2_	0.15	0.06	0.24	0.001
Dox	EtCO_2_	0.05	0.02	0.08	0.001
P	mOAAS	0.97	0.93	1.02	0.206
Dox	mOAAS	0.96	0.92	1.01	0.087

Groups are analyzed separately

*P* Placebo group, *Dox* Doxapram group, *SpO*_*2*_ peripheral oxygen saturation, *EtCO*_*2*_ end-tidal CO_2_, *mOAAS* modified observer’s assessment of alertness/sedation, *CI* Confidence interval. *B* (time) shows the slope (per min) of the modeled linear time dependence for each measured variable

**Table 3:** Mixed effects model for EtCO_2_, group, and time dependency

Variable	*B*	95% CI		*p*	Explanation
		Lower	Upper		
Intercept	3.80	3.13	0.04	*p* < 0.001	Estimated EtCO value of group P at the beginning
Group	0.44	− 0.50	1.38	0.355	Difference between groups at the beginning, group Dox vs group P
Time	0.15	0.08	0.22	*p* < 0.001	Linear time dependence (slope per min) in group P
Grp × time	− 0.10	− 0.19	− 0.01	0.034	Difference in time dependence between group Dox and group P

*EtCO*_*2*_ end-tidal CO_2_, *CI* confidence interval, meaning of B is stated in the explanation

There were no other statistically significant differences between the groups in any of the measured parameters during recovery.

There were no statistically significant differences between the groups in endoscopist and patient satisfaction (Table 1).

## Discussion

In this placebo-controlled, double-blind, and randomized study, no differences were found between placebo and doxapram regarding the alleviation of respiratory depression. It may be that the dosage used was too small to counteract the respiratory depression caused by deep propofol sedation and a larger dose might give a more favorable outcome. While the dosage for boluses of doxapram we used was in line with previous studies [6, 19], there are no studies where doxapram was used as an infusion during a procedure, only after. Therefore, we decided to use a low dosage for the infusion considering the possible analeptic effect of doxapram. The dosage is the lowest starting dose for worsening of COPD. It would be possible to increase the dose considerably as it is far from toxic levels of 130 mg/kg/day and lower than the dosage recommended for apnea of the newborn of 2–2.5 mg/kg/h [6].

It is also possible that the definition of hypoventilation used in this study was not optimal. While hypoxemia is undoubtedly a valid marker of hypoventilation, it does not differentiate a milder form of hypoventilation from normoventilation. EtCO_2_ measurement we used is not reliable either in this regard since it was measured from a nostril next to the nasal oxygen cannula. If the patient was breathing through their mouth the measured levels may be far lower than actual etCO_2_ levels. Another confounding factor is CO_2_ insufflation used by the endoscopist which is occasionally expelled by the patient causing significantly heightened measured levels of etCO_2_. Transcutaneous CO_2_ measurement could have revealed a more detailed picture of ventilatory status.

While there were no statistically significant differences in weight or drug consumption between the groups, the patients in group P weighed more and consequently received more propofol. While the dosage was weight dependent, it could influence the results as the dosage was administered according to absolute weight and not ideal or lean body weight.

Historically doxapram has been used as an analeptic drug in conjunction with both volatile and intravenous anesthetics due its stimulatory effect on the nervous system [20, 21]. Especially with regard to volatile anesthetics, this effect has been attributed to increased rate of breathing caused by the respiratory stimulant effect of the drug [22]. Since the arousal effect is also seen with intravenous anesthetics, this would suggest also a direct central nervous stimulatory effect. This arousal caused by doxapram was not seen in this study as the requirement for propofol and sedation levels were similar in both groups. As such, doxapram does not seem to impair propofol sedation.

Even though our hypothesis of less hypoxemia with doxapram was not confirmed in this study, one should note that while it was not statistically significant, there were more patients requiring additional doxapram boluses in group P than group DOX. As such, a larger trial might have yielded different results favoring doxapram. It is possible that doxapram would be more effective when given as a bolus when needed should respiratory depression occur and not as a prophylactic infusion. Perhaps a randomized placebo-controlled study in this setting would find more favorable results.

Doxapram is rather an old compound and its use has declined with the advent of newer fast acting sedatives and anesthetics. Still, it remains a quite potent respiratory stimulant that may well be considered to facilitate upper gastrointestinal endoscopic procedures in case of sedation-related respiratory depression even though it was shown be inefficient as a prophylactic measure when applied as an infusion. Naturally, sedation providers need to be aware of this option to be able to take advantage of it and research such as this may help clinicians keep in mind older drugs that are not as widely used anymore.

This study did not find doxapram to be efficient in reducing hypoxemia during deep propofol sedation for ERCP in general but that does not mean that there are not some subgroups of patients such as patients with chronic pulmonary or neurologic diseases that might gain benefit from doxapram in this setting and so further research on this topic is still warranted. One must also bear in mind that this was a quite small study with limited participants and a larger study might yield different results. Other limitations for this study are the fact that this was a single-center study, so its findings may not be applicable universally. Also, different sedation protocols may be used with doxapram for better results. After all, the sedation used in this study was a very deep sedation, close to general anesthesia and lighter levels of sedation might result in different outcomes.
